# Arthroscopic rotator cuff repair in elite rugby players

**DOI:** 10.4103/0973-6042.50876

**Published:** 2009

**Authors:** Amol Tambe, Ravi Badge, Lennard Funk

**Affiliations:** Wrightington Upper Limb Unit, Wrightington, UK

**Keywords:** Arthroscopy, contact sports, rotator cuff, rugby

## Abstract

**Background:**

Rugby is an increasingly popular collision sport. A wide spectrum of injuries can be sustained during training and match play. Rotator cuff injury is uncommon in contact sports and there is little published literature on the treatment of rotator cuff tears in rugby players.

**Aims:**

We therefore reviewed the results and functional outcomes of arthroscopic rotator cuff repair in elite rugby players.

**Materials and Methods:**

Eleven professional rugby players underwent arthroscopic rotator cuff repair at our hospital over a 2-year period. We collected data on these patients from the operative records. The patients were recalled for outcome scoring and ultrasound scans.

**Results:**

There were seven rugby league players and four rugby union players, including six internationals. Their mean age was 25.7 years. All had had a traumatic episode during match play and could not return to the game after the injury. The mean time to surgery was 5 weeks. The mean width of the cuff tear was 1.8 cm. All were full- thickness cuff tears. Associated injuries included two Bankart lesions, one bony Bankart lesion, one posterior labral tear, and two 360° labral tears. The biceps was involved in three cases. Two were debrided and a tenodesis was performed in one. Repair was with suture anchors. Following surgery, all patients underwent a supervised accelerated rehabilitation programme. The final follow-up was at 18 months (range: 6–31 months) post surgery. The Constant scores improved from 44 preoperatively to 99 at the last follow-up. The mean score at 3 months was 95. The Oxford shoulder score improved from 34 to 12, with the mean third month score being 18. The mean time taken to return to full match play at the preinjury level was 4.8 months. There were no complications in any of the patients and postoperative scans in nine patients confirmed that the repairs had healed.

**Conclusion:**

We conclude that full-thickness rotator cuff tears in the contact athlete can be addressed successfully by arthroscopic repair, with a rapid return to preinjury status.

## INTRODUCTION

Rugby is an increasingly popular contact sport. The nature of the game has changed over time with longer seasons and more intensive training which put the athlete at risk of injury. Rugby has been quoted as having the highest per player-hour rate of injury.[1] The injury rate in rugby union is 67.8 injuries per 1000 player hours, and for rugby league, 44.9 per 1000 playing hours.[2] Shoulder is the second most common site of injury and accounts for nearly 20% of all rugby injuries.[3]

If the shoulder joint is considered in isolation, a spectrum of injuries is sustained by the collision athlete. The incidence of rotator cuff injury in rugby has been quoted between 3-10%.[4]

The pathomechanics of shoulder injury have been studied extensively in relation to the throwing athlete. Rotator cuff tears have been well reported in American Football players,[5,6] but only two previous studies have looked at the surgical treatment of rotator cuff tears in rugby players.[3,7] One used open surgery only[7] and the other has not been published in the English literature.[8] Both studies included recreational athletes, as well as elite players.

We therefore investigated the results and functional outcomes of arthroscopic rotator cuff repairs in elite rugby players only over a two year period.

## MATERIALS AND METHODS

Elite rugby players undergoing a shoulder arthroscopic procedure were identified retrospectively from an electronic database. The inclusion criteria were:

1. Professional/semi-professional rugby player
2. Undergone arthroscopic rotator cuff repair for rotator cuff injury
3. A minimum follow-up of 12 months.

Over a two year period eleven players fitting the inclusion criteria were identified as our study cohort. Amongst the eleven, there were 7 Rugby league and 4 rugby union players, including 6 internationals. The mean age was 25.7 years (range= 19 to 31 years).

All patients had a pre-operative ultrasound scan, MR arthrogram or both. Operative data was collected on the dimensions of cuff tear, tear location and cuff repair technique used, number and type of anchors used as well as any associated injuries.

Patients were seen at regular intervals post-operatively and were recalled for outcome scoring and ultrasound scans. Outcomes were assessed by the Constant and Oxford scores. We also recorded the time taken to return to their previous level of play following surgery.

### Surgical technique

All procedures were carried out by the senior author. All the patients underwent arthroscopy in the beach chair position. Standard arthroscopic portals were used.

The rotator cuff tears were classified according to the Cofield Classification.[9] This is shown in Table 1. The mean size of the tear was 1.8 cm.

**Table 1:** Size of cuff tears, using the Cofield's classification

Type	Size	Number (percentage)
Small	<1 cm	3 (27)
Medium	1–3 cm	5 (46)
Large	3–5 cm	2 (18)
Massive	>5 cm	1 (9)

The mean time from injury to surgery was 5 weeks. The surgical findings and repair techniques for each patient is detailed in Table 2. The rotator cuff was mobilised in the standard fashion. The subacromial bursa and the torn edge of the rotator cuff were debrided using a combination of a soft tissue resector and radiofrequency. Subacromial decompression was not performed in these cases. The footprint was prepared and the cuff repaired using titanium or PEAK threaded suture anchors, double loaded with high strength sutures. A rotator cuff tendon to bone repair was performed in a single row configuration with a modified Mason-Allen technique[10] in all cases except three, where a double row repair was done.

**Table 2:** Surgical findings and procedures

Patient	Age	Tear size	Retraction	Repair technique	Anchor types	Attachment site	Biceps tendon	Additional findings
1	29	2 cm	Damaged but not retracted	Single bony anchor - modified Mason-Allen locking suture repair	Fastin anchor with orthocord (Mitek)	At cuff insertion	Tenodesis	Bony Bankart
2	28	2 cm	Damaged but not retracted	Single bony anchor - modified Mason-Allen locking suture repair	Fastin anchor with orthocord (Mitek)	At cuff insertion	None	360 degree labral tear, chondral injury
3	27	1 cm	Damaged but not retracted	Single bony anchor - modified Mason-Allen locking suture repair	Fastin anchor with orthocord (Mitek)	At cuff insertion	None	Reverse Bankart
4	29	2 cm	Damaged but not retracted	Single bony anchor - modified Mason-Allen locking suture repair	Fastin anchor with orthocord (Mitek)	At cuff insertion	None	Bankart, ACJ osteolysis
5	25	3 cm	Stage III - severe	Four bony anchors - double row	Spiralok with panacryl and versalok (Mitek)	Medialised	Debridement	Nil
6	24	1 cm	Damaged but not retracted	Single bony anchor - modified Mason-Allen locking suture repair	Fastin anchor with orthocord (Mitek)	At cuff insertion	None	Nil
7	19	1 cm	Damaged but not retracted	Single bony anchor - modified Mason-Allen locking suture repair	Fastin anchor with orthocord (Mitek)	At cuff insertion	None	Nil
8	25	> 5 cm	Stage III - severe	Four bony anchors - double row	Twinfix PK triple and footprint (Smith and Nephew)	At cuff insertion	None	Nil
9	26	1.5 cm	Damaged but not retracted	Single bony anchor - parachute technique	Twinfix PK triple (Smith and Nephew)	At cuff insertion	None	360 degree labral tear
10	31	3 cm	Stage II - moderate	Two bony anchors - double row	Twinfix PK triple and footprint (Smith and Nephew)	At cuff insertion	Debridement	Nil
11	22	2 cm	Damaged but not retracted	Single bony anchor - modified Mason- Allen locking suture repair	Twinfix PK triple (Smith and Nephew)	At cuff insertion	None	Bankart

Associated injuries were seen in six patients and included two Bankart lesions, one bony Bankart lesion, one posterior labral tear and two 360 degree labral tears. All were repaired at the same time as the rotator cuff repair. The biceps was involved in three cases. Two were debrided and a tenodesis performed in one. A full thickness subscapularis tear was also present in one case and was concomitantly repaired. One massive tear required medialisation to allow a tension-free repair.

Post-operatively, the patients underwent an accelerated rehabilitation programme. This has been developed by the senior author with rugby clubs and is a sports-specific programme for rugby rehabilitation. It is based on functional movement patterns and incorporates early sports-specific exercises. A sling immobilizer is used for less than one week. The first three weeks comprise of closed kinetic chain exercises which incorporate proprioception, core stability and scapula stabilizing exercises. These are done within the safe zone and progressed as tolerated. Pertubation training is introduced after three weeks and functional open chain exercises introduced. Depending on the athletes' progress, resistance exercises are introduced after approximately six weeks, along with skills training. Simulated tackling is started at about two to three months. Impact and tackle bag training is not started until the athletes have achieved satisfactory movement, strength, isokinetic and proprioceptive criteria.

## RESULTS

One hundred and twenty elite rugby players underwent arthroscopic shoulder surgery over a two-year period. Of these, 11 (9.1%) had full thickness rotator cuff tears and underwent arthroscopic repair. All of the eleven players sustained an injury during matchplay. Three players gave a convincing history of an Abduction-External rotation type impact without a dislocation, whilst one player sustained a true dislocation. Six players had sustained a direct impact injury, with their arm adducted and internally rotated at the time of impact.

All the players had pain and weakness after the injury. A common feature was pain and weakness on abduction exercises, such as dumbbell flies and shoulder press. Almost all players had a loss of confidence and none were able to return to rugby after the index injury.

The outcome score data is shown in Table 3. The mean final follow-up was 18 months (range: 12-31). The mean Constant scores improved from 44 pre-operatively to 99 post-operatively, with the strength parameter showing the most marked improvement. The mean Constant score at three months was 95. The Oxford Shoulder Score improved from 34 pre-operatively to 12 post-operatively, with a three month score of 18. The mean time of return to full match play was 4.8 months (3 to 8 months). 10 players went back to play at the same level of sport whereas one retired for personal reasons, but went on to do a heavy manual job. Patients were recalled for post-operative ultrasound scans at least 12 months post repair. Nine players returned. In all of these the ultrasound confirmed the integrity of the repair. One player underwent a repeat arthroscopy at 4 weeks post-operatively, as there was a concern about his slow response to rehabilitation. The rotator cuff repair was seen to be intact and healing. This player went on to make full functional recovery and play at international level six months later.

**Table 3:** Outcome scores and return to play

Patient	Age	Time - Inj to surg (weeks)	Injury mechanism	CS preop	CS 3 m	CS final	Final follow-up (months)	OS preop	OS 3 m	OS final	Return to play (months)	Comments
1	29	3	ABER dislocation	42	94	100	12	42	30	4	4	Played in World Cup 5 months after surgery
2	28	3	Impact	30	92	100	15	29	12	8	4	Played in World Cup 7 months after surgery
3	27	4	Impact	34	97	100	22	36	20	4	8	
4	29	7	Impact	55	80	98	30	47	16	10	6	Won player of the season following year - GB cap
5	25	5	Impact	66	85	92	27	18	17	11	No - other reasons	Retired from Rugby for other reasons, went to heavy building job
6	24	8	Not known	24	100	100	28	24	19	10	3	
7	19	5	ABER	46	96	97	18	34	17	7	5	Played international 6 months after surgery
8	25	3	Impact	22	100	100	12	42	16	12	4	scored try on first game back
9	26	5	Impact	73	96	100	17	22	18	7	5	
10	31	8	ABER	42	95	100	19	35	18	10	4	
11	22	4	ABER	46	N/A - had repeat arthroscopy	97	12	42	16	16	6	
Mean		5		44	95	99	18	34	18	9	4.8	

CS = Constant Score; OS = Oxford Score; ABER = 'Abduction and External Rotation'

## DISCUSSION

Rugby is a collision sport where significant shoulder injuries are sustained. Labral injuries are the most common and full-thickness rotator cuff tears in the young athlete are extremely uncommon.[11]

It has been postulated that rotator cuff injury starts as an aggregation of multiple microtraumatic events such as those occuring with tackles (20-30 per match), scrums (25 per match), mauls (30 per match), and lineouts (31 per match).[12] Repetitive overloading of the static shoulder stabilizers eventually cause them to fatigue and weaken. In this situation, a macrotraumatic event during training or matchplay can cause a full thickness tear in a cuff which is already 'at risk' of injury.

The shoulder in the throwing athlete has been studied in detail and the damage to the cuff as a result of 'internal impingement' and subsequent tearing is well documented in literature.[4,13,14] The mechanism of injury is repetitive hyper-abduction and external rotation. The commonest injury mechanism in our study was also an abduction and external rotation event, although this involved more trauma than an overhead athlete.

Rotator cuff lesions in the contact athlete are known to be associated with traumatic shoulder dislocation. Burkhart has described avulsion of subscapularis and the middle and inferior gleno-humeral ligaments along with a shell of bone from the lesser tuberosity in football players.[11] It is interesting to note that in our series only one out of the eleven players had a history of true shoulder dislocation. This underlines the fact that in such athletes, rotator cuff injury can occur in the absence of shoulder dislocation and hence rotator cuff injury should be suspected and ruled out in rugby athletes who sustain a significant shoulder injury without dislocation of the joint.

Goldberg *et al*, published on six elite rugby union and rugby league players with rotator cuff tears and shoulder instability.[7] They used a two stage technique, an open rotator cuff repair followed 10 weeks later by an open shoulder stabilisation. While all their six patients presented with signs and symptoms of a rotator cuff tear, four had a feeling of 'looseness' in their shoulder. Only one patient had a documented frank dislocation. At surgery, Bankhart lesion was found in one case. Anterior capsular shift was utilised to address instability. All their patients returned to contact sport at nine months with five of the six patients going back to rugby at a similar level.

There is a belief that arthroscopic procedures are not as successful as open procedures in these contact athletes.[15,16] Tibone published results of open repair in partial or full thickness cuff tears in the throwing athletes in 1986.[17] Their initial results were discouraging. Since then, arthroscopic techniques have evolved rapidly and have been used increasingly in the treatment of RCT in professional as well as amateur athletes. Flurin *et al*, report on 29 rotator cuff tears in professional Rugby players from the French Rugby championships from 1996 to 2006.[8] They used arthroscopic repair technique for partial and full-thickness cuff tears. Eighty-three percent of the patients were able to play Rugby at their preinjury competitive level after a mean of 5.5 months. Our study group had larger tear sizes and were all full thickness tears, but the results are very similar. Our series of all arthroscopic rotator cuff repairs shows that not only did the players have excellent functional recovery, they also went back to their previous level of sport early (4.8 months). We believe that this is due modern arthroscopic techniques with extremely strong sutures and anchors, with very secure repair techniques, which allow for an early sports-specific accelerated rehabilitation programme. We also believe it is essential to repair all concomitant lesions at the same time to full symptom free recovery.

In conclusion, full-thickness rotator cuff tears in the contact athlete can be addressed successfully by arthroscopic techniques. The incidence of associated injuries is high. Arthroscopic cuff repair gives good, reproducible results with rapid return to play.

## References

[1] O'Brien C (1992). Retrospective survey of rugby injuries in the Leinster Province of Ireland 1987 - 1989. Br J Sports Med.

[2] Garrawy WM, Lee AJ, Hutton SJ, Russell EB, Macleod DA (2000). Impact of professionalism on injuries in rugby union. Br J Sports Med.

[3] Bathgate A, Best JP, Craig G, Jamieson M (2002). A prospective study of injuries to elite Australian rugby union players. Br J Sports Med.

[4] Burkhart SS, Morgan CD, Kibler WB (2003). The disabled throwing shoulder: Spectrum of pathology, Part I: Pathoanatomy and biomechanics. Arthroscopy.

[5] Foulk DA, Darmelio MP, Rettig AC, Misamore G (2002). Full-thickness rotator-cuff tears in professional football players. Am J Orthop.

[6] Kaplan LD, Flanigan DC, Norwig J, Jost P, Bradley J (2005). Prevalence and variance of shoulder injuries in elite collegiate football players. Am J Sports Med.

[7] Goldberg JA, Chan KY, Best JP, Bruce WJ, Walsh W, Parry W (2003). Surgical management of large rotator cuff tears combined with instability in elite rugby football players. Br J Sports Med.

[8] Flurin PH, Guillemette C, Guillo S (2007). Traumatic rotator cuff tears in rugby players. J Traumatol Sport.

[9] Cofield RH (1982). Subscapular muscle transposition for repair of chronic rotator cuff tears. Surg Gynecol Obstet.

[10] Castagna A, Conti M, Markopoulos N, Borroni M, De Flaviis L, Giardella A (2008). Arthroscopic repair of rotator cuff tear with a modified Mason-Allen stitch: mid-term clinical and ultrasound outcomes. Knee Surg Sports Traumatol Arthroscopy.

[11] Burkhart SS, Klein JR (2004). Arthroscopic treatment of full-thickness rotator cuff tears in the athlete. Oper Tech Sports Med.

[12] (1999). International Rugby Board. Conference on the game: Proceedings, December 8.

[13] Jobe FW, Kvitne RS, Giangarra CE (1989). Shoulder pain in the overhead or throwing athlete: The relationship of anterior instability and rotator cuff impingement. Orthop Rev.

[14] Burkhart SS (2006). Internal impingement of the shoulder. Instr Course Lect.

[15] Gartsman GM, Hammerman SM (1997). Full thickness tears: arthroscopic repair. Orthop Clin North Am.

[16] Warner JJ, Goitz RJ, Irrgang JJ, Groff YJ (1997). Arthroscopic assisted rotator cuff repair: patient selection and treatment outcome. J Shoulder Elbow Surg.

[17] Tibone JE, Elrod B, Jobe FW, Kerlan RK, Carter VS, Shields CL (1986). Surgical treatment of tears of the rotator cuff in athletes. J Bone Joint Surg Am.

